# A Systematic Literature Review of Nutrition Interventions Implemented to Address Food Insecurity as a Social Determinant of Health

**DOI:** 10.3390/nu15153464

**Published:** 2023-08-05

**Authors:** Kennedy Norris, Stephanie Jilcott Pitts, Heidi Reis, Lindsey Haynes-Maslow

**Affiliations:** 1 Department of Public Health, East Carolina University, Greenville, NC 27834, USA; kennedynorris98@gmail.com; 2 Laupus Health Sciences Library, East Carolina University, Greenville, NC 27834, USA; reish21@ecu.edu; 3 Department of Health Policy and Management, University of North Carolina at Chapel Hill, Chapel Hill, NC 27599, USA; lhaynes6@email.unc.edu

**Keywords:** food insecurity, produce prescriptions, medically tailored meals, community-supported agriculture, social determinants of health

## Abstract

Background: Policy initiatives have provided funding for non-acute nutrition interventions to address food insecurity as a social determinant of health, but more research is needed to understand the outcomes of these initiatives in order to determine the areas of highest impact. Therefore, the purpose of this systematic review was to evaluate the outcomes that were assessed in three nutrition interventions (produce prescription programs, medically tailored meals, and community supported agriculture) that aim to address food insecurity as a social determinant of health, and this was undertaken in order to identify future areas of study that can heighten impact. Methods: This systematic review was performed in accordance with the Preferred Reporting Items for Systematic Reviews and Meta-Analysis (PRISMA) criteria. A list of search terms and keywords were compiled by the research team. A Boolean search was conducted from 1 January 2000 to 1 January 2023 in the PubMed advanced search database. Results: A total of 1015 articles were initially pulled from the PubMed database, and, after a screening process, 21 articles were included in our review. Nineteen of the articles focused on adult populations or families and two focused on children. The main outcomes assessed were changes in self-reported dietary intake, while a few of the articles addressed feasibility and cost-related outcomes. Conclusions: More research is needed to assess whether nutritional interventions to address food insecurity as a social determinant of health are feasible and more cost effective in the long term. Additionally, more work should be conducted in pediatric populations, which could have a robust return on investment in terms of both healthcare utilization and healthcare expenditure.

## 1. Introduction

In 2021, the United States Department of Agriculture (USDA) reported that 34 million people are food insecure in the United States [1]. A disproportionate number of children, single-parent households, racial/ethnic groups, and people from disadvantaged backgrounds suffer from low food security [1]. Food insecurity is associated with poor glycemic control [2], cardiometabolic diseases [3], and chronic disease [4]. The increase in the number of individuals with these conditions leads to higher healthcare utilization and higher spending and costs [5]. Due to the higher costs associated with food insecurity, Medicaid coverage has expanded in some states to include new and reformed nutrition services focused on alleviating food insecurity, which were not offered previously [6,7]. In particular, as a part of North Carolina’s (NC) Medicaid transformation plan (prior to the passage of Medicaid expansion in March 2023), the Healthy Opportunities Pilots (HOPs) program was initiated in March 2022 [6,7]. The aim of the HOPs is to improve health outcomes related to social determinants of health (e.g., food insecurity), thereby lowering Medicaid expenditures due to improved outcomes [8]. Social determinants of health are “conditions in which people are born, grow, live, work and age” [9], and food insecurity is an example of a social determinant of health because it is “a household-level economic and social condition of limited or uncertain access to adequate food” [10].

As an example of how addressing food insecurity can lower healthcare utilization and costs, dietary intake, BMI, and diet-related health outcomes (such as blood pressure and cholesterol) are all linked to food insecurity, insofar as those who are food insecure often have worse dietary behaviors and, thus, have higher BMI and are at increased risk of high blood pressure and cholesterol [2,3,4]. Therefore, if food insecurity is reduced, dietary intake, BMI, and other related health outcomes should also decrease, and this would result in reductions in healthcare expenditure.

Specifically, the North Carolina HOPs target food insecurity through medically tailored meal delivery, healthy food boxes, produce prescription programs, nutrition and cooking coaching/counseling, and increased links to community-based food services [6,7]. However, the outcomes associated with these three strategies, which provide tangible food (medically tailored meal delivery, healthy food boxes/ community supported agriculture, and produce prescription programs), have not been systematically evaluated or compared. Thus, we selected these three interventions as the focus of the current systematic review. For the purposes of this review, a produce prescription program was defined as a program that is implemented to impact the dietary intake of fresh fruits and vegetables by providing prescriptions for them in the form of a voucher, cash, or an allotment of a card [11]. Programs that implement medically tailored meals are those that tailor specific, pre-packaged food in order to meet nutrients that are medically required in order to address certain chronic conditions through food [12]. Community-supported agriculture (CSA) is a type of direct-to-consumer marketing that is a partnership between local farms and customers (“members”), who purchase a CSA share in return for regular food deliveries from that farm. The food and the farm thereby become tied to the community, where mutual care of the land is distributed, and where the goods that are gathered are shared [13].

In addition to the North Carolina HOPs, the USDA has authorized the creation of the Gus Schumacher Nutrition Incentive Program (GusNIP), which was formerly known as the “Food Insecurity Nutrition Incentive Program [11]. The GusNIP provides funding for eligible organizations which implement and evaluate projects that provide incentives to low-income participants in order to increase their purchase of fruits and vegetables, which they do through nutrition incentive programs and produce prescription programs. Thus, the purpose of our systematic review was to evaluate the outcomes that have previously been assessed for these specific nutrition interventions, and the focus was on the North Carolina HOPs (medically tailored meal delivery, healthy food boxes/ community supported agriculture, and produce prescription programs) that aim to address food insecurity as a social determinant of health. The goal of our paper is to identify future areas of study in order to heighten the impact of HOPs.

## 2. Materials and Methods

Literature search strategy: This systematic literature review was conducted in accordance with the Preferred Reporting Items for Systematic Reviews and Meta-Analysis (PRISMA) [12,14]. A Boolean search was developed in Medline via the PubMed interface by librarian HR in consultation with KN, using keywords related to food insecurity, cardiometabolic diseases, nutrition interventions, and study types. No publication date filter was used, and the search was run from inception to January 2023, with 1015 results. The full search strategy can be seen in the ECU institutional repository entry at http://hdl.handle.net/10342/12701 (accessed on 4 May 2023). A total of 1015 articles were screened based on the inclusion and exclusion criteria described below.

Inclusion and exclusion criteria: The focus of this review was peer-reviewed publications that focused on the effectiveness of three specific non-clinical food interventions on food insecurity or chronic conditions. Dissertations, white papers, review articles, and abstract-only papers were excluded. This review used only quantitative results as the primary outcome measure. The articles included had to have a population from a developed country. Study outcomes had to include either dietary outcomes, such as increased intake of fruits and vegetables or whole grains, or food insecurity, body mass index, health-related outcomes (such as improvement in HbA1c levels in diabetic participants, blood pressure levels in hypertensive participants), feasibility outcomes, or cost-related outcomes. The papers included had to have recruited and reported on data collected from participants from a public health system or a health care system.

**Figure 1:**
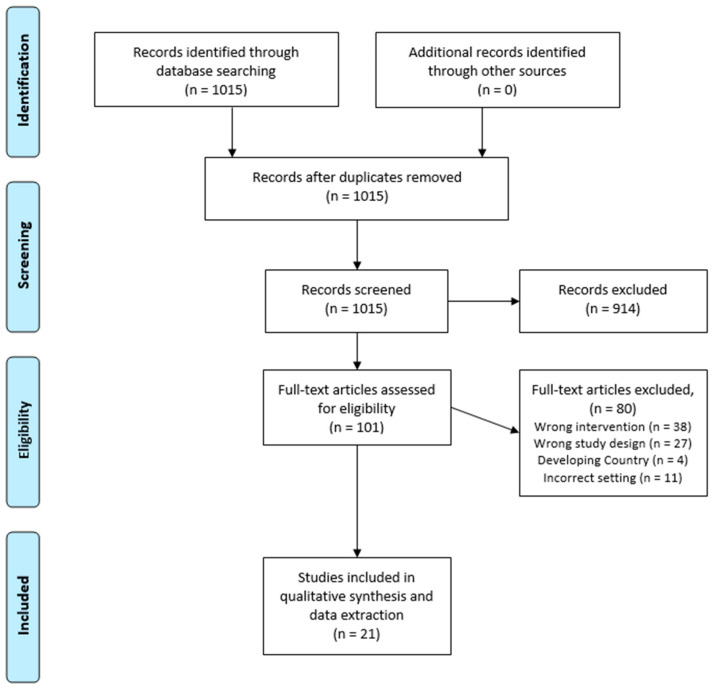
PRISMA flowchart of the reviewed studies.

Data Extraction and Analysis: Using Covidence, (https://www.covidence.org/, access on 8 May 2023) an online screening operator tool, a title and abstract screening was performed first (BernardDeckerPublicLibraries.edu, 2023). The primary author screened the initial 1015 initial articles (See Figure 1). After the first screening, 914 articles were deemed irrelevant to the primary focus of this review. Independently, a two-author screening was conducted for the full-text screening portion of the remaining 101 articles. All of the conflicts were resolved during a designated meeting between the primary and secondary author. A total of 27 articles were selected for the extraction screen. During extraction, 6 articles were additionally excluded for inclusion in the final qualitative synthesis as they were found to not be directly linked with a health clinic or a health department as the referring entity for program participants. The following information was extracted: setting, length of study, type of study, number of participants, eligibility criteria, program type, dosage, and population demographic characteristics (e.g., mean age, race/ethnicity, socioeconomic status). The following outcomes were extracted: changes in BMI, dietary intake, food insecurity, condition improvements, feasibility, and cost-related outcomes. For the purpose of this review, feasibility refers to retention rates in the studies, successful attendance at nutrition education sessions (if applicable), redemption rates for vouchers (if applicable), and overall ease of use for participants. Cost-related outcomes could refer to cost effectiveness, the dollar amount per outcome achieved, or cost savings. The authors of the study used SNAP-Ed’s definition of direct nutrition education. Indirect nutrition education [15], defined as “the distribution or display of information and resources, including any mass communications, public events (such as health fairs), and materials distribution, which involve no participation interaction with an instructor or multimedia”, was not considered as nutrition education. Additionally, following SNAP-Ed definitions and guidelines, the study’s authors did not include diabetes self-management education (DSME) as nutrition education, since nutrition education focuses on the prevention diet-related chronic diseases whilst DSME focuses on managing an already diagnosed condition.

## 3. Results

Table 1 shows an overview of the included studies, and Table 2 provides more details on all of the studies included. A total of 21 studies were reviewed, including 13 (61.9%) food or produce prescription program studies [16,17,18,19,20,21,22,23,24,25,26,27,28,29], 4 (19.0%) medically tailored meal (MTM) program studies [5,30,31,32], and 4 (19.0%) community-supported agriculture (CSA) studies [33,34,35]. Fifteen studies (71.4%) focused on adult populations and four (19.0%) focused primarily focused on families and pediatric populations. Only two (9.5%) of the studies focused on children. Nineteen (90.5%) of the articles were implemented in the United States in various states, and the remaining two studies were implemented in Canada and Australia. Over half of the study designs (n = 13, 61.9%) were quasi-experimental with a pre-post design, while only two (9.5%) were randomized controlled trials. Additionally, eight of the studies (38.1%) provided direct nutrition education to their participants, although one study specifically noted the lack of attendance at classes due to accessibility issues [33].

The demographics of the sample populations varied, but the populations generally included a racially and ethnically diverse sample. Additionally, 16 of the studies had a majority female participant population, while 3 did not report the gender of the participants. Fourteen of the studies required that study participants were food insecure, uninsured, or using some type of federal food assistance program, such as SNAP or WIC, in order to be eligible to participate. A total of 15 of the 21 studies required that program participants be diagnosed with at least one chronic condition or be at risk of a chronic condition in order to be eligible to participate. The chronic conditions that participants had to be diagnosed with or at risk of consisted of prediabetes, type 2 diabetes, a cardiometabolic disorder, or being overweight/obese.

In terms of the outcomes assessed (Table 3) in the 21 studies, 10 studies (47.6%) assessed changes in BMI, 17 examined changes in dietary intake (81.0%), 13 (61.9%) examined improvement in food security status, 14 (66.7%) measured improvements in participants’ chronic conditions, 12 (57.1%) assessed feasibility-related outcomes, and 4 (19.0%) assessed cost-related outcomes. Of the 10 studies that assessed changes in BMI, 2 studies [27,32] found a statistically significant reduction in BMI, 7 [17,18,26,28,30,31,35] found non-statistically significant changes, and 1 [33] found an increase in BMI. For dietary intake in the 17 studies (81.0%), 9 (42.9%) found an increase in fruit and/or vegetable intake [19,20,21,22,23,24,25,28,32], and 5 (23.8%) found no statistically significant changes in this intake [19,26,31,33,34]. Two studies (9.5%) found an increase in whole grain consumption [24,33]. For studies that used the HEI (n = 3), all of them found statistically significant improvements in the HEI score [27,30,35]. A total of 12 out of the 13 studies examining food insecurity found improvements in the food security status of the participants [16,18,19,21,22,24,27,29,30,31,32,35].

A total of 14 out of the 21 studies measured other health condition improvements, ranging from hemoglobin A1c, hypertension, waist circumference, mental and physical health, cholesterol, and diabetes management. The results for these outcomes were mixed, and most were not statistically significant. However, four studies found reductions in blood pressure [18,22,29,35], four found decreases in hemoglobin A1c [22,26,31,33], two found reductions in waist circumference [22,29], and one found improvements in cholesterol [27]. In terms of feasibility, 12 of the studies examined aspects of feasibility [16,17,18,19,24,26,27,31,32,33,34,35] using the main measurements of redemption or distribution rates, adherence to nutrition education when offered, food waste, and participant satisfaction. For studies that tracked cost-related outcomes [5,16,26,27], these included cost per redemption, total cost distributed to participants, avoided produce costs to participants, and estimated reduction in medical costs per month.

## 4. Discussion

In this literature review, we examined the outcomes that have been assessed in three specific nutrition interventions, all of which aimed to address food insecurity as a social determinant of health in order to identify the areas of improvement that are needed to advance the field (n = 21 studies). The majority of papers included in this comprehensive literature review are produce or food prescription programs (n = 13, 61.9%). There should be more studies dedicated toward evaluating the effectiveness of MTMs, CSAs, and other non-clinically based nutrition interventions. In addition, most of the studies were focused on adult populations, and more work is needed in pediatric populations as children have not already developed chronic diseases, and, moreover, intervening among children would probably also provide a robust return on investment. When children have healthy habits and can maintain those habits throughout their lifespan [36], this will likely decrease healthcare utilization and expenditure related to chronic disease management among older adult populations.

The majority of studies utilized a quasi-experimental pre-post design. There should be additional studies using randomized controlled trials as the study design. There are qualified, dedicated health departments or federally qualified health centers that could be tested as potential referring entities, and this would open more intervention availability among many communities. Eight studies included a nutrition education component. In some cases, these nutrition education sessions were poorly attended and under-utilized. More work is needed to encourage participation in nutrition education sessions so that participants can increase their knowledge and confidence regarding how to use provided produce.

Overall, the most frequently measured outcome was changes in dietary intake among the study participants. The second most frequently measured outcome was status changes regarding food security. Because the three interventions examined in this review have the ultimate goal of reducing food insecurity and improving nutrition security, future evaluations of similar interventions should include a measure of food insecurity and nutrition security outcomes. Furthermore, only one study included in this review [27] used objective measures of dietary intake. Future studies should evaluate the impact of these three nutrition interventions on objective measures of diet, which are not subject to recall biases and social desirability [37].

One outcome that was used to assess feasibility was a measure of redemption rate, but redemption rates were measured differently across the articles. For example, Abel et al. [17] examined the total number of vouchers redeemed divided by the total number of vouchers distributed, while Aiyer et al. [16] examined the average number of prescriptions redeemed per participant and the mean number of times the prescriptions were redeemed. Additional studies could determine the most salient measures of redemption rates in order to ensure that future studies define redemption rates similarly across studies.

Other reviews have been conducted in order to assess how clinics or healthcare organizations are offering access to healthy foods. In one review paper written by Veldheer et al., 8876 articles were screened and a total of 44 manuscripts were retained for inclusion in their review [38]. The review by Veldheer et al. utilized only papers that were clinic-based, whereas this current review included articles in both a clinical and community health center. The inclusion of public health departments or centers could possibly have provided a better overview of the population for each particular area due to more marginalized and disadvantaged populations receiving care at community health sites. Veldheer et al. did not assess whether the studies were cost effective or the feasibility of the studies that were included [38]. It is important to assess cost benefits from each study so that input versus output spending can be analyzed, replicated, and improved.

Bhat et al. [39] conducted a meta-analysis to examine healthy food prescription programs and their impact on dietary behaviors, BMI, systolic and diastolic blood pressure, HbA1c, and blood lipids. They found a 22% increase in FV intake, a BMI decrease of 0.6 kg/m^2^, and a HbA1c decrease of 0.8% [39]. Bhat et al. also noted the need for large, randomized controlled trials to examine the efficacy of healthy food prescription programs [39].

Currently, 41 states have adopted Medicaid expansion [40]. Some states, such as North Carolina, have begun implementing and evaluating programs that have the potential to reduce healthcare costs. The results of Medicaid expansion programs, such as PPP, MTM, and CSAs, will lend insight into whether these programs improve diet, overall health and well-being, and healthcare cost savings, thereby reducing hospital readmission rates and also reducing dependency on the use of emergency services. If the results of these evaluations are promising, Medicaid and other insurance companies could benefit from investing in food delivery programs due to their ability to reduce healthcare expenditure. While this review demonstrated that few papers evaluated cost-related outcome data (healthcare expenditures), this may be due to the complexities of measurement. Since private and public insurance companies negotiate pricing with healthcare organizations, it is difficult to quantify healthcare expenditures, especially when examining costs across different healthcare entities and different geographic regions/states. Therefore, going forward, having a more straightforward measure of healthcare utilization may be more insightful for researchers who examine the cost effectiveness of food programs.

The USDA authorized GusNIP provides funding to provide incentives to low-income participants through nutrition incentive programs and produce prescription programs. While data from some of these projects have been evaluated, others are currently being evaluated in order to assess feasibility, diet-related outcomes, and reduction in healthcare utilization. This systematic literature review can help inform future GusNIP request for applications or even provide information for potential grantees on lessons learned and what has been most effective to date.

This paper has both strengths and limitations. One strength is that two independent authors screened full-text articles and extracted data from all of the included papers. Discrepancies were discussed at a designated time between the two authors, and a consensus was reached regarding each paper or disagreement. A librarian was consulted with in order conduct the Boolean search from PubMed advanced database. However, there are some limitations to this review. The PubMed database was the only search engine used in this literature review, and more search engines could have yielded more or different results. Additionally, the primary author screened titles and abstracts, but for more validated results, the two independent authors could both have screened all items from the beginning of the process. Furthermore, there was no formal definition of developed countries, and, thus, some papers could have been inadvertently not included. Two of the included articles [16,23] were food vs. produce prescription programs, but we included these due to their specific focus on produce when describing the intervention. In the future, it will be important to compare the outcomes of food versus produce prescription programs. Lastly, a quality assessment of each article was not conducted by the authors.

## 5. Conclusions

To inform evidence-based policy supporting funding for nutrition interventions to address food insecurity as a social determinant of health, more research is still needed. Few of the studies examined in the current review included any cost-related outcomes. Thus, there need to be more studies analyzing the cost effectiveness of interventions to address food security as a social determinant of health. Since GusNIP has been funding nutrition incentive programs, we hope that grantees will continue to publish their results. More specifically, in the 2018 farm bill, GusNIP changed its focus to also include healthcare costs and utilization rates. However, this data has been difficult to evaluate. Going forward, there should be clear distinctions between the average cost of an acute care stay or medical visit tailored for chronic conditions and food insecurity compared to the cost-benefit analysis of these non-acute care-based nutrition interventions. In the same capacity, studies that show feasibility will only encourage replication in more areas where the services are needed.

Overall, there were many positive results of these nutrition interventions, and they should result in reductions in healthcare costs and improved population health. If additional studies are conducted and demonstrate similar positive results in terms of health outcomes and cost reductions, additional investments in these programs will be warranted.

## Figures and Tables

**Table 1:** Condensed outcomes from this systematic literature review of nutrition interventions implemented to address food insecurity as a social determinant of health.

Characteristic	Number of Studies (%)
Type of intervention	
CSA	4 (19.0%)
MTM	4 (19.0%)
Produce Rx	13 (61.9%)
Provided Nutrition Education	8 (38.1%)
Outcomes	
BMI	10 (47.6%)
Dietary intake	17 (81.0%)
Food security changes	13 (61.9%)
Disease Improvements	14 (66.7%)
Feasibility	12 (57.1%)
Cost-related outcomes	4 (19.0%)
Age group	
Adults	15 (71.4%)
Families	4 (19.0%)
Children	2 (9.5%)

**Table 2:** Characteristics of studies included in this systematic literature review of nutrition interventions implemented to address food insecurity as a social determinant of health.

Citation	Program Type	Setting	Eligibility Criteria	N	Study Design and Dates of Data Collection	Intervention Description	Nutrition Education Included?	Race/Ethnicity, Gender	Mean Age (SD), in Years	Socioeconomic Indicators	Adults, Families, or Children
Aiyer et al., 2019 [16]	Produce RX	Two school-based clinics and one federally qualified health center, Harris County, North Pasadena, Texas	An age of 18 or older, food insecure, resided in one of three targeted zip codes	242 enrolled, 172 redeemed vouchers	Quasi-experimental pre-post; September 2016–May 2017	6-month prescription eligible for redemption every 2 weeks; 12 redemptions and 30 lbs. of produce per redemption	Yes	79.7% Hispanic, 3.5% African American, 79.1% female	47.3 (13.6)		Adult patients and parents of pediatric patients
Abel et al., 2022 [17]	Produce RX	Irving Medical Center in Northern Manhattan, New York	English- and Spanish-speaking patients, June and November 2019	242	Retrospective, cross-sectional study; baseline, June–November 2019; follow-up, November 2019–February 2020	6 months; $10 for food secure or $20 for food insecure patients for prescription for fruits and vegetables from local greenmarkets	No	48% of redeemers and 37% of non-redeemers spoke Spanish; gender not assessed	Mean age not provided; 30% pediatric and 70% adult	Redeemers: 56% SNAP, 30% WIC. Non-Redeemers: 42% SNAP, 47.5% WIC	Patients and parents/guardians of pediatric patients
Cook et al., 2021 [18]	Produce RX	Six primary care and community-based sites across Atlanta, Athens, and Augusta, Georgia	SNAP eligible or screened positive for food insecurity, and diagnosed or at risk of ≥1 diet-related chronic conditions	122 program graduates, with 63 lost to follow-up	Quasi-experimental pre-post; April–August 2017	6-month group-based nutrition and hands-on cooking education along with nutrition subsidies for fresh produce worth $1 per family member per day, redeemable weekly	Yes	78.7% African American, 77.0% female	Mean age not provided; 50% were aged 45–64 years	Program graduates: 61.7% any public assistance, 57% SNAP, 4.7% WIC	Adults
Forbes et al., 2019 [20]	Produce RX	Medical Center in Hershey, Pennsylvania, and two local farmers markets	Families or individuals aged from 5 to 75 years, patients identified by physician as being at-risk of chronic illness, with difficulty obtaining fruits and vegetables	10 enrolled, and 9 completed the program	Quasi-experimental pre-post; Fall 2015	6 weeks; $40 per visit to spend on produce at a farmer’s market for a total of four farmer’s market visits	Yes	66.7% African American, 22.2% white, 11.1% unknown 55.6% female	Mean age not provided	66.7% had a total family income below $40,000	Adults and children
Heasley et al., 2021 [21]	Produce RX	Two community health center locations in Guelph, Ontario, Canada	Food insecure ≥ 1 cardio-metabolic conditions or micronutrient deficiency	Total of 60; 36 responded to follow-up surveys	Quasi-experimental, pre/post; September 2019–June 2020	12 weeks; 12 vouchers to the Community Food Market, valued at $10/person with a household maximum of $50/household	No	Not assessed	47.2 (12.5)	52% receiving disability support; 40% with income between $10,000–19,999	Adults
Kerr et al., 2020 [22]	Produce RX	A diabetes research Institute, Santa Barbara County, California	Age of 18 years or older, Type 2 Diabetes for at least 6 months or at-risk of Type 2 Diabetes	159	Quasi-experimental, pre/post; February 2019–March 2020	10 weeks; 10 weekly prescriptions with 21 servings/week of fresh vegetables	No	75% Hispanic, 20% white, 2.5% African American, 2% Asian, 0.5% Native American; 76.7% female	52.5 (13.2)	35% uninsured	Adults
Oliveira et al., 2021 [23]	Produce RX	Health Center, South Miami, Florida	Food insecure, BMI > 40, or BMI 35 with ≥2 chronic conditions	4 (10 were initially recruited)	Prospective case report; no dates provided	4 months; biweekly packages of fresh fruits and vegetables	Yes	25% African American, 25% Hispanic, 50% Haitian American 100% female	Not provided	100% uninsured	Adults
Saxe-Custack, et al., 2021 [24]	Produce RX	Children’s Clinic in Flint, Michigan	Caregiver whose child was between ages of 8 and 18 years, food insecure, English-speaking	Total of 122 caregiver-child dyads (244 participants)	Quasi-experimental, pre/post; August 2018–March 2020	12 months; $15 prescription for fruits and vegetables during each clinic visit	No	Child’s race: 63% African American, 27% white, 10% not reported/other; Caregiver’s race: 59% African American, 29% white, 12% other/not reported Child’s gender: 52% female; Caregiver gender: 93% female	Child’s mean age: 12.42 (2.78), caregiver mean age 39.94 (10.28)	Caregiver: 37% HS degree or less, 43% some college/technical degree, 19% Bachelor’s degree or more	Children
Slagel et al., 2023 [25]	Produce RX	Nurses Clinic, Athens, Georgia	Age of 18 years or older, SNAP eligible or otherwise underserved, diagnosis of ≥1 diet-related chronic conditions	24 (16 intervention and 8 control participants)	Non-randomized controlled trial; June–December 2017	6 months; participants received a produce prescription every month, worth $1/day per household member, redeemed once per week at the local farmers’ market	Yes	57.4% white, 37.5% Hispanic 79.6% female	47.5 (11.3)	90.7% uninsured, 98.1% annual household income ≤ $25,000	Adults
Veldheer et al., 2021 [26]	Produce RX	Hospital in Reading, Pennsylvania	Age of 18 years or older, Type 2 diabetes, HbA1c > 7.0%, BMI of ≥25	97	Quasi-experimental with a single arm pre/post; June 2018–May 2019	7 months; the monthly dollar amount equivalent to $1/household member/day for 28 days (range, $28–$140/month), with vouchers provided in $2 increments	No	81.4% Hispanic, 12.4% white, 6.2% African American; 66% female	53.8 (11.6)	65.9% SNAP recipients, 89.4% food insecure	Adults
Wu et al., 2022 [27]	Produce RX	Hospital in Sydney, Australia	Age of 18 years or older, Type 2 diabetes, food insecure	50 (49 completed 6-week and 46 completed 12-week dietary assessments)	Quasi-experimental, pre/post; November 2020–October 2021	12 weeks; prescriptions for food were designed for 2 meals/day, 5 days per week including fruits, vegetables, beans/legumes, whole grains, plain milks, cheese, and plant-based fats	Yes	African or Middle Eastern 2%, Asian 12%, European 34%, Oceanian 42%, Peoples of the Americas 10%; 46% female	63.0 (9.0)	60% total annual household income < $25,948	Adults
Xie et al., 2021 [28]	Produce RX	Federally qualified health center, outpatient clinic serving low-income patients and two organizations in Durham, North Carolina	Age of 18 years or older, SNAP recipients, had grocery store loyalty card	699	Prospective cohort study; grocery store data, April 2018–June 2019; Electronic Health Record data, November 2017–June 2019	12 months; $40 monthly voucher was given to participants to spend at a grocery store chain	No	81.4% African American, 11.6% white, 5.3% Hispanic, 1.7% other; 72.4% female	58.5	86.8% Medicaid, Medicare, were uninsured, or insurance coverage other than private insurance	Adults
York et al., 2020 [29]	Produce RX	Local social services, Latino-focused community organizations, and existing diabetes programs	Hispanic participants with self-reported type 2 diabetes diagnosis	21	Quasi-experimental, pre/post; no dates provided	12 weeks; medically prescribed organic vegetables provided weekly	No	100% Hispanic; 91% female	56 (11.1)	Not reported	Adults
Berkowitz et al., 2019 [5]	MTM	Community health center in Boston, Massachusetts	Age of 18 years or older, reside in the targeted area, captured in the Massachusetts All-Payer Claims Database at least 360 days before the study	1020	Retrospective cohort study; MA All-Payer Claims Database from 2011–2015; study conducted December 2016–January 2019.	Mean duration of receipt of meals was 12.4 (10.6) months; weekly delivery of 10 ready-to-consume meals tailored to specific medical needs under supervision of a dietitian	No	23.8% White, 13.5% African American, 4.5% Hispanic, 1.7% Multiracial/other, 56.6%, information not provided 5; 53.3% female	52.7 (14.5)	9.7% living in poverty, 20.9% Medicare recipients, 56.3% Medicaid recipients	Adults
Berkowitz et al., 2018 [30]	MTM	Primary care networks and physicians in Eastern Massachusetts	Age of 18 years or older, diagnosed with type 2 diabetes, A1c > 8% in the last year, food insecure, reside in targeted area	42	Randomized, cross-over trial; June 2015–July 2017	24 weeks: 12 weeks of once-a week delivery of 10 refrigerated and/or frozen meals, followed by 12-week wash-out period	No	Intermediate: 25% African American, 50% white, 20% Hispanic, 5% other; Delayed: 27% African American, 59% white, 14% Hispanic, 0% other; Female 65%; immediate, 73% delayed	Immediate, 57.66 (12.25), 59.21 (13.11)	Immediate: 108.56 of Federal Poverty Level (FPL); Delayed: 170.38 FPL (median income level was 140% of the FPL)	Adults
Kempainen et al., 2023 [31]	MTM	Healthcare organization and community-based organization, Minneapolis, Minnesota	Type 2 Diabetes diagnosis, age of 21 to 70 years, receiving care at Hennepin healthcare, screened positive for food insecurity	281	Randomized, controlled prospective pilot study; no dates provided	24 weeks; every 2 weeks, home-delivered meal boxes (30–33 pounds) tailored to nutritional needs and ethnic food preferences	No	67% African American, 21% White, 9% Native American, 3% Hispanic, 7% Pacific Islander/Asian or other; Gender not assessed	55.6	50% disabled, 13% employed part-time, 7% employed full-time, 13% unemployed, 11% retired, and 6% home-maker/other.	Adults
Palar et al., 2017 [32]	MTM	San Francisco Bay area, California	Current client, living with HIV or type 2 diabetes, English- or Spanish-speaking, age of 18 years or older, low-income under 300% of FPL	52	Quasi-experimental pre/post; April 2014–June 2015	6 months; meals and snacks picked up 2/week to provide 100% of daily caloric requirements tailored to meet nutritional guidelines	No	28.9% African American, 28.9% White, 21.2% Hispanic, 9.62% Native American, 1.92%, Asian/Pacific Islander, 9.62% other/mixed; 34.9% female	57.2 (9.8)	17.3% employed, 21.6% on SNAP; less than high school/GED 13.5%, high school/GED 17.3%	Adults
Fischer et al., 2022 [19]	CSA	Outpatient clinics, Washington DC	Adults with young children, ages of 0–5 years, food insecure, diet-related chronic disease risk factor	25 families	Quasi-experimental pre-post; recruitment in December 2020.	12 months; families received a 12-month supply of bi-weekly deliveries of fresh produce; produce delivery included approximately 8 lbs. of seasonal, locally-sourced fresh fruits and vegetables	Yes	100% African American; 100% female	29.9 (5.8)	40% had less than $10,000 per year for income	Families with children
Tester and Leak, 2021 [33]	CSA	Children’s Hospital and Research Center, Oakland, California	8–17 years of age, confirmed diagnosis of prediabetes, low SES households (enrolled in public insurance), ≥1 primary care giver, residing in targeted zip codes	47	Quasi-experimental, pre/post; enrollment January–June 2017 and follow-up May–October 2017.	16 weeks; CSA shares had approximately 1/2 serving of vegetables per person/day; bi-weekly deliveries included two 15-oz cans or 1 lbs. of dried beans/legumes and at least 1-oz-eq/day of whole grains per person	Yes	Children: 26% African American, 66% Hispanic, 2% Asian, 6% Mixed/other; Caregivers: 2% White, 24% African American, 72% Hispanic, 2% Asian; Children: 47% female; Caregivers: 96% female	Children: 12.9 (2.4); Adults: 43.0 (9.8)	59% of households had an annual household income ≤ $30,000, received SNAP (52.5%), and were food-insecure (55%)	Children
Izumi et al., 2017 [34]	CSA	Multnomah County Health Department, Portland, Oregon	Patient of FQHC; had to speak Spanish or English	25	Quasi-experimental, pre/post; intervention began June 2015	23 weeks; subsidized CSA share; most participants paid $5/week and the remainder of the costs were funded through a grant	No.	Race: 4% African American, 56% white, and 24% other; Race/ethnicity: 40% Hispanic; 92% female	64% between 35 and 60 years of age	64% on SNAP; Highschool degree or less 24%, some college 44%, college degree or more 24%	Adults
Berkowitz et al., 2019 [35]	CSA	Health Center, Franklin County, Massachusetts	18 or older, BMI of >25 kg/m^2^, seen at the community center or lived in surrounding area	122	Randomized controlled trial; May 2017–December 2018	24 weeks; received $300 to buy a CSA share from a local farm	No	90.2% White, 2.5% African American; 1.6% Hispanic, 5.7%, Asian/multi/other; 82% Female	50.3 (13.6)	Median income = 146% of the FPL, 39.2% received SNAP benefits, 36.7% food insecure	Adults

**Table 3:** Outcomes assessed (“Not reported” indicates that the change in that variable was not reported in the publication).

Citation	BMI	Dietary Intake	Food Insecurity	Condition Improvements	Feasibility	Cost-Related Outcomes
Aiyer et al., 2019 [16]	Not reported	99% reported eating most or all of the provided food	Food insecurity decreased significantly (94.1% decrease)	Not reported	73.1% participation rate of total screened candidates, 172 Rx redeemed on average, participants redeemed 6.5 times of the available 12 redemptions	$12.20 per family per redemption
Abel et al., 2022 [17]	No statistically significant changes in BMI.	Not reported	Patients who were food insecure at baseline were more likely to redeem their prescription than those not food insecure at baseline	Those who redeemed their vouchers were more likely to have elevated hemoglobin A1c than non-redeemers	Of the 2368 prescriptions distributed from June to November 2019, 49.3% were redeemed	Not reported
Cook et al., 2021 [18]	No statistically significant changes in BMI.	Not reported	42% had increased food security	Program Graduates had lower diastolic blood pressure, smaller waist circumferences, and lower baseline triglycerides. Unadjusted estimates indicate that diastolic blood pressure was modestly but significantly reduced by 0.73 mmHg for every visit completed	Participant retention from the first to the third visit was 64.0%, while retention from the first to the last visit was 22.6%	Not reported
Fischer et al., 2022 [19]	Not reported	Children’s fruit and vegetable intake increased: 43% increase in average daily fruit intake (*p* = 0.02) and 29% increase in average daily vegetable intake (*p* = 0.21)	Food insecurity decreased from baseline to post-intervention, but the decrease was not statistically significant	Not reported	77.5% reported produce was used or frozen for future use, 80% were very or completely satisfied with produce variety, attendance at classes was 63.1% per participant, and retention was 60% at 12 months	Not reported
Forbes et al., 2019 [20]	Not reported	Daily fresh fruit consumption increased from 37.5% before the program to 62.5% after the program. Green vegetable intake of one serving/week increased from 62.5% to 87.5%, and orange-colored vegetable intake increased from 38% to 87.5%	Not reported	Not reported	Not reported	Not reported
Heasley et al., 2021 [21]	Not reported	Increased F/V intake by two servings extra per day on average. Weekly intake of fruits increased from 4.7 to 8.5 (*p* = 0.05) and other vegetables increased from 3.5 to 5.2 (*p* = 0.02)	At follow-up, 26 respondents improved their adult food security scores (74%), six households had poorer scores (17%), and three (8.6%) had no change compared to baseline scores	No changes in self-reported mental or physical health	Not reported	Not reported
Kerr et al., 2020 [22]	Not reported	Drop in intake of tortillas and soda, and frequency of vegetable intake increased significantly, with 50% of 120 participants consuming vegetables at least once per day compared with 15% at baseline (*p* < 0.0001)	Proportion of low or very low food security at enrollment was 35% and dropped to 13% after 3 months (*p* < 0.001).	Waist circumference reduced (−0.77, *p* = 0.022), systolic BP reduced (−2.42, *p* = 0.037), weight reduced (−0.4 kg, *p* = 0.029), and HbA1c decreased by −0.35% (*p* = 0.009).	Not reported	Not reported
Oliveira et al., 2021 [23]	Not reported	Increase in weekly fruit consumption, including increase in fruit servings per day	Not reported	Not reported	Not reported	Not reported
Saxe-Custack, et al., 2021 [24]	Not reported	Increase in mean child-reported daily intake of vegetables (*p* = 0.001), whole grains (*p* = 0.001), fiber (*p* = 0.008), and dairy (*p* < 0.001)	Household food security improved (*p* < 0.001) from baseline (1.96 ± 2.20) to follow-up (0.87 ± 1.25); child-reported food security improved from baseline (*p* = 0.01)	Not reported	5953/7827 eligible patients received prescriptions for a 76% distribution rate	Not reported
Slagel et al., 2023 [25]	Not reported	Intervention group reported increased fruit and vegetable intake, 0.81 (SD = 0.91) serving/day, versus control group, −0.25 (SD = 0.99) serving/day (*p* = 0.02)	Not reported	Not reported	Not reported	Not reported
Veldheer et al., 2021 [26]	No statistically significant changes in BMI	There was a 0.49 times/day increase in combined F&V intake at follow-up, but this was not statistically significant	Not reported	There was a −1.3% decrease (*p* < 0.001) in HbA1c	DSME retention was 62%; the voucher redemption rate was 83.4% and the voucher redemption rate-intention to treat was 53%. Participants attended an average of 3.6 nutrition sessions and all 7 visits were completed by 45.4% of participants	On average, participants received a total of $353 in vouchers and redeemed a total of $295
Wu et al., 2022 [27]	Statistically significant reductions in BMI (−0.67) (*p* = 0.002)	Mean AHEI scores increased 52.3 to 65.2 pre/post intervention. Seven components of the AHEI increased from pre/post intervention. There were no changes in the biomarkers assessed (Vitamin C, zinc, and magnesium)	Food insecurity decreased from 82% at baseline to 0% at 12-week follow-up	Improvements in mean total cholesterol (−0.28), LDL (−0.23), HDL (0.06), and total HDL cholesterol ratios (−0.48) (*p* < 0.001)	All who participated had at least one dietitian consultation, and 74% participated in all consultations	Not reported
Xie et al., 2021 [28]	No statistically significant changes in BMI	“Frequent Spenders” had increased fruit and vegetable variety and the number of unique FV items in a month. Higher program utilization was correlated with higher FV purchasing. Frequent Spender status was correlated with higher monthly FV spending	Not reported	Program utilization was not associated with diabetes diagnosis, systolic blood pressure, or Emergency Department visits	Not reported	Not reported
York et al., 2020 [29]	Not reported	Not reported	Food insecurity improved in 12/21 (57.1%) of participants	Reduction in systolic and diastolic blood pressure (*p* = 0.03 and 0.01, respectively). A total of 14 (67%) lost weight (median weight loss = 1.9 pounds), and waist circumference decreased in 9/19 (47.4%) responders (median = 1.5 inches)	Not reported	Average retail cost was $31.33 per week per participant
Berkowitz et al., 2019 [5]	Not reported	Not reported	Not reported	Significantly fewer in-patient admissions (incidence rate = 0.51) and fewer skilled nursing facility admissions (incidence rate ratio = 0.28)	Not reported	Mean monthly costs would have been $3838 vs. $4591 if no one had been encouraged into treatment. Medical cost reduction was estimated at $712 (95% CI, $1930 lower to $505 higher) per month
Berkowitz et al., 2018 [30]	No statistically significant changes in BMI.	Mean HEI score while participants were receiving the MTM was 71.3 (SD 7.5), while mean HEI score when not receiving the meals was 39.9 (SD 7.8) (difference 31.4 points, *p* < 0.0001), with improvements in almost all sub-categories of the HEI scores	42% reporting food insecurity during “on-meal” vs. 62% during “off-meal” period (*p* = 0.047)	Patient-reported reductions in hypoglycemia, with 47% reporting hypoglycemia while receiving the MTM vs. 64% while not receiving the MTM (*p* = 0.03)	Not reported	Not reported
Kempainen et al., 2023 [31]	No statistically significant changes in BMI	No statistically significant changes in fruit or vegetable intake	At baseline, 100% of participants were food insecure, while at follow-up, 87% of the control group and 78% of the treatment group were food insecure (*p* = 0.07)	Hb1Ac for control at baseline vs. follow-up = 7.89 vs. 7.78; for intervention at baseline vs. follow-up = 7.99 vs. 7.64, *p* = 0.46	93% satisfaction rate; 89% reported assistance in managing their diabetes; 89% either liked the food, used the food, and found the recipes helpful; and 98% of deliveries were made	Not reported
Palar et al., 2017 [32]	BMI decreased from 31.2 to 30.1 (*p* = 0.08). Among those with type 2 diabetes, BMI decreased from 36.1 to 34.8 (*p* = 0.035)	Frequency of consuming fatty foods decreased from 3.19 vs. 2.21 times per day (*p* = 0.003); FV intake increased 1.85 to 2.34 times per day (*p* = 0.011)	Very low food security affected 59.6% of participants at baseline vs. 11.5% at follow-up. High food security was 9.62% at baseline and 53.9% at follow-up	Among those with HIV, adherence to ARV therapy increased from 47% to 70%. Diabetes management improved. At follow-up participants reported fewer depressive symptoms (7.58 vs. 5.84, *p* = 0.028). Trends toward fewer hospitalizations and ED visits	Adherence to food pick-up was 93%. 78.9% reported eating all or most of the intervention food, and 90.4% reported throwing away intervention food at some point.	Not reported
Tester et al., 2021 [33]	BMI increased 0.5 mg/kg^2^ (*p* < 0.05) though the change in BMI z-score was not statistically significant	Whole grain consumption increased from 1.7 to 2.5 oz.-eq./day (*p* < 0.001). Total vegetable intake was unchanged from baseline to follow-up	Not reported	At follow-up, there was a statistically significant 12% increase in serum triglycerides; mean HbA1c was 5.62%, which was significantly lower than the eligibility HbA1c, at the initial study visit (*p* < 0.001). There were no statistically significant changes in other outcomes (fasting glucose, insulin, HOMA-IR, and HDL and LDL cholesterol)	At follow-up, 97% of participants reported having the cooking education binder given to them at the beginning of the study; 60% prepared some of the recipes; 96% received study text messages and 45% reported they or someone in their household watched the cooking videos. 30% said they or someone in the household had prepared a food-based recipe based on what they learned	Not reported
Izumi et al., 2017 [34]	Not reported	At baseline, 25% ate ≥ 2 cups/day of vegetables, versus 50% at follow-up (*p* = 0.38)	Not reported	Not reported	56% reported almost no waste, 33% reported ≥ 2 items wasted, and 11% reported 1 item wasted	Not reported
Berkowitz et al., 2019 [35]	No statistically significant changes in BMI. However, weight decreased 1.56 (*p* = 0.17) and BMI (−0.43, *p* = 0.44)	HEI scores increased comparing intervention (60.2) to control (55.9), *p* = 0.03; improved scores for total vegetables (4.2 vs. 3.7, *p* = 0.008), total fruit (3.2 vs. 2.2, *p* < 0.0001), whole fruit (3.1 vs. 2.4, *p* = 0.007), and lower consumption of empty calories (15.1 vs. 13.4, *p* = 0.01)	Food insecurity prevalence decreased from 42% to 32% in the control group and from 31% to 11% in the intervention group (RR 0.68, 0.48, 0.96, *p* = 0.03)	No statistically significant reductions in blood pressure and HbA1c, but statistically significant decreases in diastolic blood pressure	79% of participants picked up their weekly CSA share	Not reported

## Data Availability

No new data were created.
